# A New Omics Data Resource of *Pleurocybella* *porrigens* for Gene Discovery

**DOI:** 10.1371/journal.pone.0069681

**Published:** 2013-07-23

**Authors:** Tomohiro Suzuki, Kaori Igarashi, Hideo Dohra, Takumi Someya, Tomoyuki Takano, Kiyonori Harada, Saori Omae, Hirofumi Hirai, Kentaro Yano, Hirokazu Kawagishi

**Affiliations:** 1 Graduate School of Science and Technology, Shizuoka University, Suruga-ku, Shizuoka, Japan; 2 Bioinformatics Laboratory, School of Agriculture, Meiji University, Kawasaki, Japan; 3 Institute for Genetic Research and Biotechnology, Shizuoka University, Suruga-ku, Shizuoka, Japan; 4 Department of Applied Biological Chemistry, Faculty of Agriculture, Shizuoka University, Suruga-ku, Shizuoka, Japan; Technical University of Denmark, Denmark

## Abstract

**Background:**

*Pleurocybella* *porrigens* is a mushroom-forming fungus, which has been consumed as a traditional food in Japan. In 2004, 55 people were poisoned by eating the mushroom and 17 people among them died of acute encephalopathy. Since then, the Japanese government has been alerting Japanese people to take precautions against eating the *P* *. porrigens* mushroom. Unfortunately, despite efforts, the molecular mechanism of the encephalopathy remains elusive. The genome and transcriptome sequence data of *P* *. porrigens* and the related species, however, are not stored in the public database. To gain the omics data in *P* *. porrigens* , we sequenced genome and transcriptome of its fruiting bodies and mycelia by next generation sequencing.

**Methodology/Principal Findings:**

Short read sequences of genomic DNAs and mRNAs in *P* *. porrigens* were generated by Illumina Genome Analyzer. Genome short reads were *de novo* assembled into scaffolds using Velvet. Comparisons of genome signatures among Agaricales showed that *P* *. porrigens* has a unique genome signature. Transcriptome sequences were assembled into contigs (unigenes). Biological functions of unigenes were predicted by Gene Ontology and KEGG pathway analyses. The majority of unigenes would be novel genes without significant counterparts in the public omics databases.

**Conclusions:**

Functional analyses of unigenes present the existence of numerous novel genes in the basidiomycetes division. The results mean that the omics information such as genome, transcriptome and metabolome in basidiomycetes is short in the current databases. The large-scale omics information on *P* *. porrigens* , provided from this research, will give a new data resource for gene discovery in basidiomycetes.

## Introduction

In eukarya, fungus is a huge group consisting of various microorganisms such as yeasts, molds and mushrooms. To date, about 69,000 fungal species have been identified and reported. However, the total number of fungal species in the world is estimated at around 1.5 million [1]. In mushroom-forming fungi, which have economic values as edible and medicinal resources for human [2,3], 21,000 species have been identified so far [4]. Hawksworth *et al.* (2001) has inferred that around 140,000 species of mushrooms-forming fungi exist on Earth [5]. Identification of the characteristics of various mushrooms species facilitates a more efficient and effective utilization as nutrient and medicinal resources [6]. It also helps to understand the structure and dynamics of complex ecological systems.

A basidiomycete *Pleurocybella* *porrigens* (division : Basidiomycota, order : Agaricales) is a mushroom-forming fungus, which has been consumed as a traditional food in Japan. In 2004, however, 55 people were poisoned by eating the mushroom and 17 people among them died of acute encephalopathy. Since then, the Japanese government has been alerting Japanese people to take precautions against eating the *P* *. porrigens* mushroom. Ever since the food-poisoning incident, we have been trying to understand the molecular mechanism for the acute encephalopathy and have reported the isolation and characterization of a lectin and unusual amino acids from the mushroom, which might be related to the accident [7–9]. There are also some papers concerning the mushroom reported by other researchers [10–12]. However, the real truth of the molecular mechanism for the acute encephalopathy still remains elusive.

Conventionally, isolations of toxins produced from mushrooms have been studied using bioassays measuring their intrinsic toxicity. For example, Matsuura et al. (2009) have isolated cycloprop-2-ene carboxylic acid from the lethal mushroom *Russula subnigricans* [13]. This compound is lethal at 2.5 mg/kg by oral administration against mice. We have also reported the isolation and characterization of a family of isolectins (*Boletus venenatus* lectins, BVLs) from the diarrheagenic mushroom *B* *. venenatus* [14]. Oral administration of BVLs caused diarrhea in rats. However, all the compounds obtained from the mushroom *P* *. porrigens* did not cause acute encephalopathy in animals. This might be due to unkown mechanism involved in causing toxicity than that of usual toxicity mentioned above. The omics data such as genome and transcriptome data improve our abilities to analyze biological functions of toxicity from *P* *. porrigens* .

Public database for omics data on fungi ‘The Fungal Genome Initiative’ (http://www.broad.mit.edu/annotation/fgi/) at the Broad Institute provides genome sequences information for more than 50 fungal genomes [15]. However, the genome and transcriptome sequence data of *P* *. porrigens* and the related species are not stored in the public database. To gain the omics data and knowledge in *P* *. porrigens* , sequenced genome and transcriptome data of fruiting bodies and mycelia by next generation sequencing is reported here. The computational analysis was also performed to predict biological functions of each transcript. The large-scale omics data in *P* *. porrigens* open new avenues to advance our understanding of fungal species.

## Results and Discussion

### Sequencing of genomic DNAs and mRNAs

Short read sequences (100 bp in length) of genomic DNAs and mRNAs in *P* *. porrigens* were generated by Illumina Genome Analyzer (Table 1). The short read sequences from genomic DNAs contain 60,919,280 paired-end (PE) sequence reads (30,459,640 pairs) and 83,048,614 mate-paired (MP) reads (41,524,307 pairs). PE reads from mRNAs were also generated; 75,071,884 reads (37,535,942 pairs) in the fruiting bodies and 69,747,206 reads (34,873,603 pairs) in the mycelia.

**Table 1:** The numbers of sequencing reads.

Library	Number of raw reads	Number of high-quality reads
Genome (PE reads)	60,919,280	50,292,262
Genome (MP reads)	83,048,614	59,947,894
Transcriptome of fruiting bodies (PE reads)	75,071,884	51,405,754
Transcriptome of mycelia (PE reads)	69,747,206	50,806,810

We obtained high-quality read sequences by removing regions with low quality scores in fastq files (quality scores < 20) and reads containing one or more ambiguous nucleotide site(s) from the raw Illumina sequencing data. Genome sequence reads, 50,292,262 (82.6%) PE reads and 59,947,894 (72.2%) MP reads which were pre-processed were employed for further analysis. 51,405,754 (68.5%) transcriptome sequence reads of the fruiting bodies and 50,806,810 (72.8%) transcriptome sequence reads of mycelia which were pre-processed were also employed for further analysis (Table 1).

### *De novo *assembly

We assembled the high-quality genomic DNA sequences into scaffolds. We used the program Velvet [16] to assemble reads, since Velvet investigates and eliminates the contamination of MP reads (see the Velvet manual in detail). The assembling of PE reads by Velvet provided contigs; the largest N50 contig length of 931 bp under k=81. With the contigs and MP reads (3 kb insert size library), we obtained scaffolds by assembling (Table 2A). The largest N50 length (1598 bp) was obtained under k=87. The range of the scaffold lengths was 173 to 22,324 bp (the average length was 1,032 bp).

**Table 2:** Assembly summary.

**A. Genome**		
Number of scaffolds	31,164	
Total size of scaffolds (bp)	32,149,440	
N50 (bp)	1,598	
Average scaffold length (bp)	1,032	
Maximum scaffold length (bp)	22,324	
Minimum scaffold length (bp)	173	
**B. Transcriptome**		
	Fruiting bodies	Mycelia
Number of contigs	45,390	26,216
Total size of contigs (bp)	29,504,308	11,748,163
N50 (bp)	1,069	633
Average contig length (bp)	650	448
Maximum contig length (bp)	9,955	8,825
Minimum contig length (bp)	100	100

The high-quality short reads from mRNAs were assembled into unigenes (contigs, a non-redundant sequence set) by the program Oases [17]. To avoid confusion in the terminology, the contigs obtained from transcriptome sequences are referred to as ‘unigenes’. We found the best assembly to be at k=45 (fruiting bodies) and k=49 (mycelia), as it resulted in the largest N50 length of 1069 bp and 633 bp, respectively. We obtained 45,390 and 26,216 unigenes in the fruiting bodies and the mycelia (Table 2B).

### Comparison of genome signature between *P. porrigens* and other basidiomycetes and ascomycetes

Comparison on tetranucleotide-based genomic signatures among genomes has been a powerful tool to assess the similarity in the genome sequences [18,19]. For classification of species (genomes) according to similarities of profiles (genome signatures), a statistical method on the basis of corresponding analysis (CA) is efficient and effective. Phylogenetic analysis (phylogenic tree) is also useful to assess the evolutionary difference between two species. However, it is not easy to assess the differences among more than two species at once. CA provides plots of species in a low-dimensional projection (space), and the distance between plots (species) directly indicates the evolutionary difference. Two species have theoretically more similar characteristic in their genome signatures, as the distance between plots converges to zero. From the statistical point of view, CA can easily identify the degrees of similarities (differences) among multiple species at once. The method is shown in a reference (Nishida H, et al. 2012) [20], which introduced the similar comparisons of genome signatures among 89 bacterial species by using CA method.

Plots of species in the low-dimensional space (Figure S1) and distances between plots (species) (Table S1) obtained from CA permit us to understand the similarities in genome signatures. The CA results show that the genome signature of *P. porrigens* was the most similar to those of *Cronartiumquercuum* and *Melampsoralaricis-populina* . *P* *. porrigens* belongs to the order Agaricales (red symbols in Figure S1), whereas *C* *. quercuum* and *M* *. laricis-populina* belong to the Pucciniales (green symbols in Figure S1). By contrast, the genome signature of *P* *. porrigens* was not close to those of the same order Agaricals.

The CA results also show the vast genome diversity among families Tricholomataceae and Agaricaceae in the order Agaricales (Figure S1). *Laccaria* *bicolor* *,* *Gymnopusluxiurians* and *P* *. porrigens* belong to the same family Tricholomataceae. *Agaricus bisporus* var *bisporus* belongs to the family Agaricaceae. Beyond the family classifications, *L* *. bicolor* and *G* *. luxiurians* show more similarity to *A* *. bisporus* rather than *P* *. porrigens* . These results indicate that *P* *. porrigens* has a unique genome signature from those of other Agaricales.

### Functional annotations for *P* *. porrigens* unigenes

**Figure 1:**
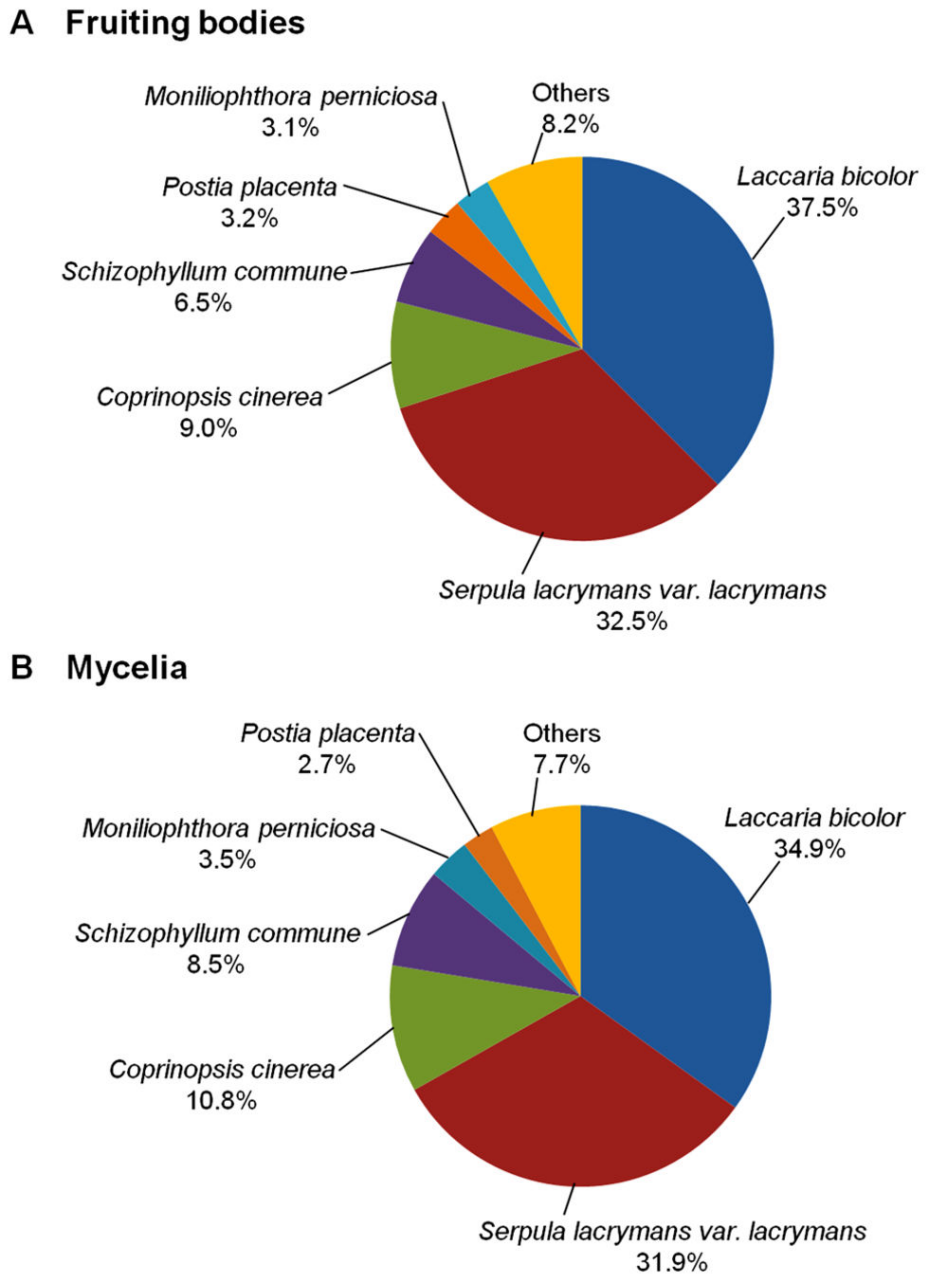
The frequency distributions of species having the significantly homologous sequence with *P. porrigens*. Species distributions of the top BLASTX hits for unigene sets in (A) fruiting bodies and (B) mycelia.

To predict the biological functions of *P* *. porrigens* transcripts (unigenes), unigene sets of the fruiting bodies and the mycelia were compared against the NCBI non-redundant (nr) database by the BLASTX program. For the fruiting bodies unigenes and the mycelia unigenes, 2,219 and 2,834 have significantly homologous sequences in the nr database, respectively (Table 3). The majority (92%) of the significantly homologous sequences in the nr database originated from related fungal species, predominantly *L* *. bicolor* and *Serpula* *lacrymans* var. *lacrymans* (Figure 1). The distributions of species having the significantly homologous sequences with *P* *. porrigens* were nearly same between fruiting bodies and mycelia (*p*=0.022 from chi-square test). For the majority (76%) of the significantly homologous sequences, the biological functions are still unknown (data not shown), since their functional annotations (descriptions) have not been clearly described yet (e.g., ‘unknown protein’, ‘hypothetical protein’ and ‘expressed protein’).

**Table 3:** Unigenes annotation summary.

	Number of unigenes	
	Fruiting bodies	Mycelia
Total number of unigenes	45,390	26,216
Unigenes having significant similar sequences in the nr database	2,219	2,834
Unigenes assigned with GO slim terms	11,101	5,570
Unigenes assigned with KO IDs	9,085	5,251

**Figure 2:**
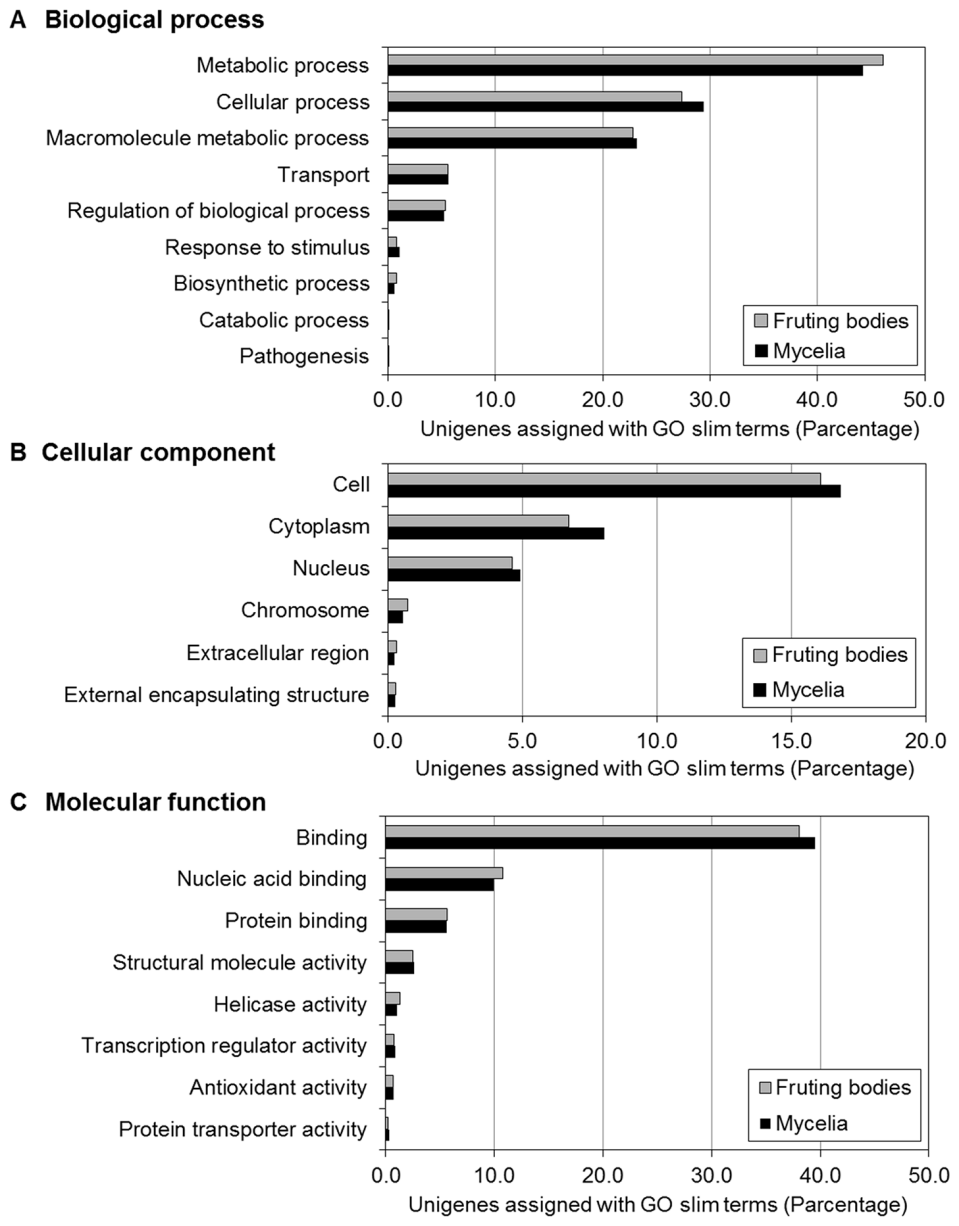
Distributions of GO slim terms assigned to the unigenes. (A) Biological process, (B) cellular component, (C) molecular function.

Among all the unigenes, 11,101 (24.5%) and 5,570 (21.2%) unigenes in the fruiting bodies and the mycelia were assigned with one or more Gene Ontology (GO) slim term(s), respectively. Against the unigenes assigned with a GO slim term(s), distributions of the GO slim terms are shown for the three GO categories (biological processes, cellular components, molecular functions) in Figure 2. The distributions of GO slim terms in the fruiting bodies and the mycelia were nearly the same as each other. We statistically analyzed the similarity of distribution between the fruiting bodies unigenes and the mycelia unigenes by chi-square test. The statistical test did not show any significant differences among the distribution patterns in any of the three GO categories. The significance probabilities were 0.021, 0.115 and 0.255 in biological process, cellular components and molecular function, respectively.

In the three GO categories, although the statistical test above did not show highly significant differences, the ratios of frequencies in GO slim terms between the fruiting bodies and the mycelia are high in some GO slim terms (Figure 2). For the category of biological process, the term ‘metabolic process’ was over-represented in the fruiting bodies (Figure 2A). It seems that metabolisms of the fruiting bodies are slightly more active than that of the mycelia. For the category of cellular component, the GO slim terms ‘extracellular region’ and ‘chromosome’ were over-represented in the fruiting bodies (Figure 2B). Extracellular matrix plays mainly a structural role, it has been reported that extracellular matrix influences most aspects of cellular behavior including migration, differentiation, survival and signaling [21–23]. Chromosomal proteins include structural elements of the chromatin, which promote transcription by modifying chromatin conformation [24]. These results suggest that the genes related in development and transcription are activated during morphogenesis in the fruiting bodies. For the category of molecular function, the GO slim terms ‘nucleic acid binding’ and ‘helicase activity’ were over-represented in the fruiting bodies (Figure 2C). Nucleic acid binding proteins are involved in transcription and translation events [25]. Helicases are involved in genome stability and meiotic recombination through DNA replication, DNA repair, and DNA recombination in all organisms [26]. These results imply that genes related with such as metabolism, cell behavior, DNA remodeling and so on are activated in the fruiting bodies.

### Experimental analysis for fruiting bodies and mycelia

To detect genes showing different gene expression levels between the fruiting bodies and the mycelia, we investigated expression levels by read per exon kilobase per million (RPKM) measurement and semi-quantitative reverse transcription (RT)-PCR. When unigenes from the fruiting bodies and the mycelia show high sequence similarity with each other (identity ≥ 90% and coverage ≥ 50%), the unigene pair was considered to be originated from the same gene. The expression ratios between the fruiting bodies and the mycelia were calculated by comparing RPKM of each unigene pair. Unigene pairs showing more than 2 fold changes were selected as up/down-regulated gene. As a result, 666 and 2,304 unigenes were found to be up-regulated in the fruiting bodies and the mycelia, respectively, and 3,136 unigenes were almost equally expressed in both of the fruiting bodies and the mycelia.

**Figure 3:**
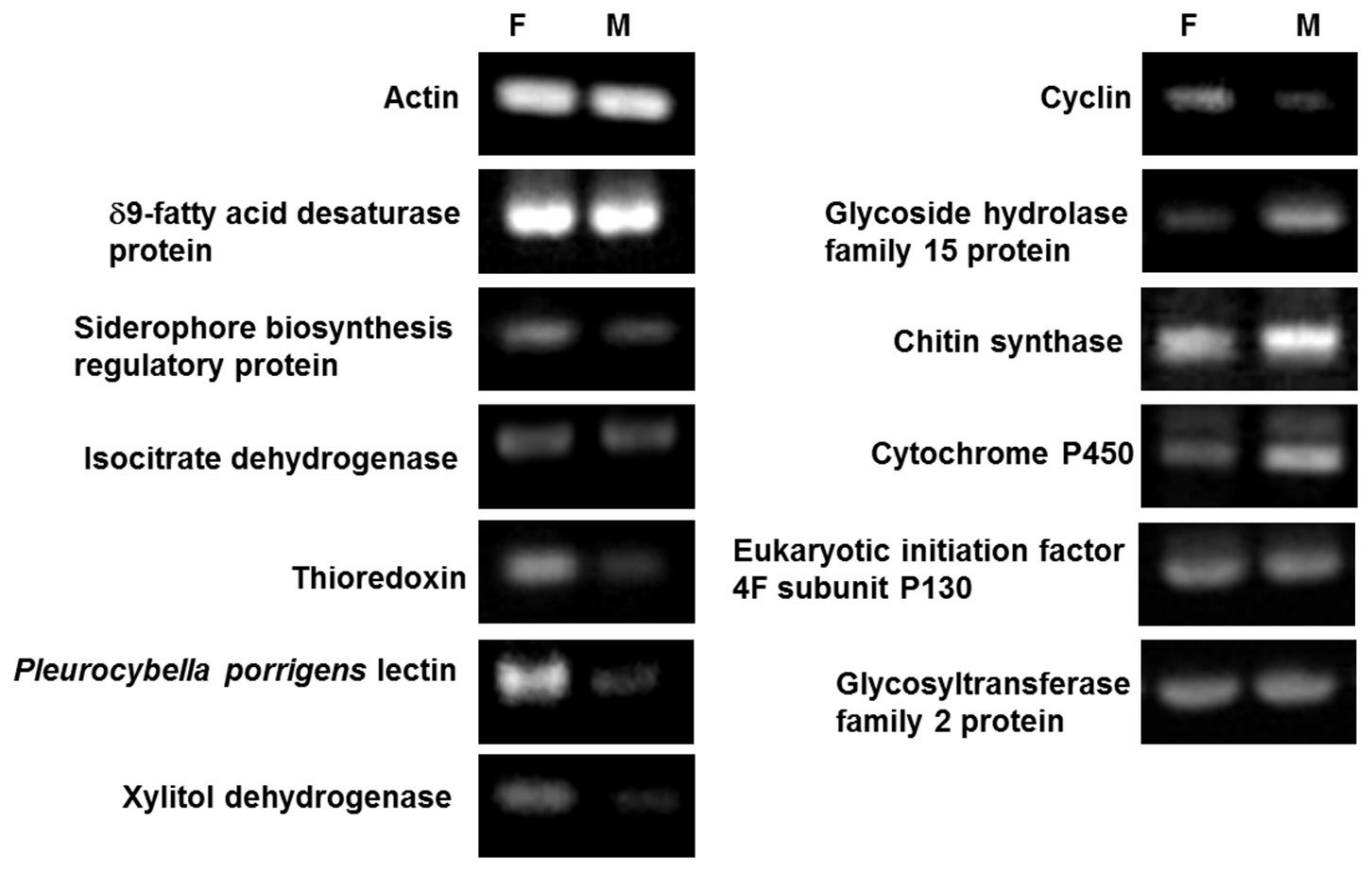
Semi-quantitative RT-PCR analysis. RT-PCR analysis of the expression of 13 genes, including an internal control: Actin, in fruiting bodies (F) and mycelia (M) of *P* *. porrigens* . Primer sequences and PCR product sizes are given in Table 4.

To validate gene expression ratios (ratios of RPKM), we performed RT-PCR for 12 genes. The 12 genes have homologous sequences in the NCBI nr database (Table S2). According to the RPKM measurement, among the 12 genes, four unigenes showed nearly the similar expression levels between the fruiting bodies and the mycelia (the ratio between 0.5–2.0), but three unigenes showed high expression levels in the fruiting bodies only. The other five unigenes showed low expression levels in the fruiting bodies. The expression levels (RPKM) were supported by the results of RT-PCR for nine unigenes showing expected sizes (Figure 3). However, for 3 out of 12 unigenes, the expression levels were inconsistent between the results of RPKM and RT-PCR. In our approach, however, the parameters in assembling and mapping were strictly determined by previous pilot tests.

The cause for the inconsistencies in expression levels between RPKM and RT-PCR were examined. First, the numbers of reads mapped into each unigene were checked. For the 12 unigenes, which were performed RT-PCR for the validation, the numbers of reads mapped into a unigene were more than median value except a unigene of *Pleurocybella* *porrigens* lectin in mycelia (Table S3). These results indicate that the numbers of reads are sufficient to evaluate gene expression levels. Subsequently, the effect of the existence of multi-mapped reads in counting reads as expression levels were investigated. The multi-mapped reads were mapped into two or more unigenes. They were used to be counted for evaluating expression levels of unigenes. Figure S2 shows the numbers of reads mapped into a unigene(s), containing uniquely mapped reads and multi-mapped reads. For the three genes showing inconsistency in expression levels between RPKM and RT-PCR, the numbers of multi-mapped reads are considerably higher than those of uniquely mapped reads in the fruiting bodies or the mycelia at least. The numerous multi-mapped reads indicate the existences of highly homologous unigenes for the three genes. Using BLAST (identity ≥ 90% and coverage ≥ 80%) search against the three genes, homologous genes were examined. As a result, one unigene, thioredoxin, has three and one highly homologous unigenes in the fruiting bodies and the mycelia, respectively. For two other genes, eukaryotic initiation factor 4F subunit P130 and glycosyltransferase family 2 protein, have also five and three highly homologous unigenes in the fruiting bodies, respectively. The existence of homologs unigenes and multi-mapped reads would make it hard to count expression levels accurately.

### Metabolic pathway analysis for unigenes

The 9,085 fruiting bodies unigenes and 5,251 mycelia unigenes were assigned with the Kyoto Encyclopedia of Genes and Genomes (KEGG) orthology (KO) identifiers (IDs) (Table 3). Among these unigenes, 555 fruiting bodies unigenes and 514 mycelia unigenes were assigned to metabolic pathways. We also identified enzymes, which seems to be related with unigenes, in the category of BRITE hierarchy of primary and secondary metabolism. For primary metabolism, the numbers of unigenes were two prominent functional hierarchy ‘amino acid metabolism’ and ‘carbohydrate metabolism’ (Figure S3 A). In secondary metabolism, one functional hierarchy ‘terpenoid backbone biosynthesis’ was considerably over-represented (Figure S3B).

We focused our attention on terpenoid backbone biosynthesis (Figure S4). The map of terpenoid backbone biosynthesis, farnesyl diphosphate synthase (EC 2.5.1.1/EC 2.5.1.10) is known to have an important role in the regulation of isoprenoid biosynthesis [27]. We observed a unigene annotated as the farnesyl diphosphate synthase in both the fruiting bodies and the mycelia. Furthermore, we observed a unigene annotated as a mevalonate kinase (EC 2.7.1.36) in the triterpenes biosynthesis pathway (Figure S4). We discovered this unigene in the mycelia is a fusion gene. The fusion gene has been reported in *Ganderma* *lucidum* [28]. The unigene detected here has the mevalonate kinase domain at the C-terminus and the cystathionine β-lyase domain at the N-terminus. This fusion gene might have a novel function and structure, and further study is required to characterize this enzyme found in fungus.

## Conclusion

In this study, we comprehensively investigated the biological functions of the genome and transcriptome in *P* *. porrigens* by next-generation sequencing technologies. With the large-scale sequence data from genomic DNAs and mRNAs, we comprehensively predicted gene expression patterns between the fruiting bodies and the mycelia. In addition, the biological functions for unigenes (transcripts) were inferred with GO slims and metabolic pathways.

The total length of all *P* *. porrigens* scaffolds by Velvet suggests the genome size of around 32 Mb. The *P* *. porrigens* genome obtained here consists of 31,164 scaffolds (N50 scaffold size of about 1.6 kb). An enormous number of short scaffolds obtained here do not provide sufficient information to get the complete genome DNA sequences in *P* *. porrigens* . However, the scaffold information is useful to estimate the genome size of *P* *. porrigens* , since the scaffolds must be composed of the partial genome sequences. In addition, the Illumina sequencing for the *P* *. porrigens* genome yielded a total of about 4.2 Gb of high-quality PE reads. This implies that the mean read depth of PE reads would be 35 to 140, assuming the *P* *. porrigens* genome size is of 30 to 120 Mb. The depth is appropriate for assembling of the genome sequences (fragments) [29–31]. Although the primary purpose behind this research was not the complete genome sequencing of *P* *. porrigens* , the additional genome sequencing data including such as long insert library or long read library helps us to release more precise and complete genome sequences for future studies.

Our large-scale computational analysis suggests the genome size of *P* *. porrigens* may be almost identical to the five Agaricals, *A* *. bisporus* , *Amanita* *muscaria* *,* *Hebeloma* *cylindrosporum* , *Pleurotus ostreatus* and *Coprinopsis* *cinerea* . The five Agaricals have the genome sizes around 31 to 36 Mb according to the databases in DOE Joint Genome Institute (http://www.jgi.doe.gov/). The total length of all *P* *. porrigens* scaffolds is almost the same with the genome sizes of the five Agaricals.

CA shows the genome signature of *P* *. porrigens* was the most similar to those of *C* *. quercuum* and *M* *. laricis-populina* . The genome signature of *P* *. porrigens* was close to the order Pucciniales rather than the same order Agaricales. The genome signatures of *C* *. quercuum* and *M* *. laricis-populina* are also similar. These results imply that these three organisms may conserve the similar DNA sequence patterns. The sequence similarity searches show highly homologous genes between *P* *. porrigens* and *L* *. bicolor* . However, the genome sizes are apparently different among the two organisms. The genome size of *L* *. bicolor* is approximately twice as large as those of *P* *. porrigens* . While the genome sizes in *P* *. porrigens* , *A* *. bisporus* , *A. muscaria* *,* *H* *. cylindrosporum* , *P. ostreatus and* *C* *. cinerea* are nearly the same, the genome signatures of those organisms are not similar. The various genome sizes and genome signatures among these organisms indicate the vast diversity in Agaricales genomes.

Functional analyses of unigenes illuminate the presence of numerous novel genes in basidiomycetes. The sequence similarity searches show that about 90% of unigenes have no significant counterpart in the NCBI nr database. Around 20% of the unigenes could be assigned with the GO slims and KEGG pathways. The results also indicate that the omics information such as genome, transcriptome and metabolome in basidiomycetes is short in the current databases. We expect the large-scale omics data of genome and transcriptome of *P* *. porrigens* presented here will play a significant role as a new data mining resource. However, more omics data from various species (genomes) in basidiomycetes should be collected and stored in the databases. Development of bioinformatics infrastructure is required to effectively and efficiently facilitate the elucidation of the gene functions and biological mechanisms.

## Materials and Methods

### Sample preparation for DNA sequencing

Fruiting bodies of *P* *. porrigens* were collected at Narusawa village, Yamanashi Prefecture, Japan. It was stored at -80 ^°^C in a freezer. No specific permits were required for the described field studies, as the sampling locations were not privately owned or protected in any way. Furthermore these field studies did not involve endangered or protected species. Mycelia of *P* *. porrigens* were provided by Miyagi Prefectural Forestry Technology Institute. For RNA isolation, the mycelia were grown in SMY liquid broth (1% sucrose, 1% malt extract and 0.4% yeast extract) at 25 ^°^C.

For genomic DNA sequencing, total genomic DNA was extracted from the fruiting bodies using the Qiagen DNeasy Plant Mini Kit (Qiagen) according to the manufacturer’s recommendations. All DNA samples were quantified using PicoGreen dsDNA Quantification Reagent (Invitrogen) according to manufacturer’s recommendations. Structural integrity of DNA was checked by gel electrophoresis.

For RNA sequencing, total RNA was extracted from the fruiting bodies and the mycelia by using RNeasy Mini Kit (Qiagen). The quality and quantity of each RNA sample were assessed as described previously [32]. Agarose gel electrophoresis and OD260/OD280 ratio were used for assessing quality of total RNA.

### Library preparation and Illumina sequencing

The Illumina library was prepared according to the manufacturer’s instructions. The library was purified with Qiaquick DNA purification kit (Qiagen). The size selected cDNA was made blunt ended with End Repair Enzyme in the presence of 2.5 mM dNTPs and 10 mM ATP (Illumina). Adenine nucleotide was added to the 3’ ends of the blunt ended cDNA with Klenow fragment (3’ to 5’ exominus) in the presence of 1 mM dATP by incubating at 37 ^°^C for 30 minutes. The double stranded cDNA with adenine on its ends was ligated with adapters (Illumina) using T4 DNA ligase at room temperature for 15 minutes. Subsequently, the cDNA was amplified with two adapter primers (Illumina) with initial denaturing step at 98 ^°^C for 30 seconds, followed by 15 cycles at 98 ^°^C for 10 seconds, 65 ^°^C for 30 seconds, 72 ^°^C for 30 seconds with a final extension cycle at 72 ^°^C for 5 minutes. The PCR products were purified with Qiaquick PCR purification kit. The products were gel-extracted and used for sequencing using Illumina Genome Analyzer.

Short read sequence data (100 bp read length) were obtained using Illumina Genome Analyzer. Genomic DNA sequencing generated PE reads with insert lengths of 300 bp and MP reads with insert length of 3 kb. The PE RNA-seq sequencing with insert lengths of 300 bp was also performed for total RNA of fruiting bodies and mycelia. The sequencing results are archived in the DDBJ Sequence Read Archive (DRA) database (accession number: DRA000925). Furthermore, the sequence data and functional annotation data generated in this study are available from http://bioinf.mind.meiji.ac.jp/P_porrigens/.

### Pre-processing of raw short read sequences and assembly

Among raw short read sequences, high-quality reads were selected for the further analysis. Low-quality regions with the quality value < 20 in fastq files were trimmed. Reads less than 5 bp were removed. Reads containing one or more ambiguous nucleotide site(s), shown by “.” in the sequence data, were also removed.

Genome short reads were *de novo* assembled using Velvet (version 1.1.04; http://www.ebi.ac.uk/~zerbino/velvet/) [16]. PE reads from genomic DNAs were assembled into contigs. Then, scaffolds were built from the contigs and MP reads. To detect optimum k-mer size which provides the largest N50, we performed Velvet with some k-mer sizes (43 to 89 mers).

Transcriptome sequences from the fruiting bodies and the mycelia were assembled into unigenes by the program Oases (version 0.1.21; http://www.ebi.ac.uk/~zerbino/oases/) [17]. To detect optimum k-mer size which provides the largest N50, we performed Oases with some k-mer sizes (43 to 89 mers).

Velvet and Oases were performed on a CentOS5.5 server (32 cores and 1 Tb memory).

### Comparisons of genome signatures between P. porrigens and other basidiomycetes and ascomycetes

The genome sequence data of the 22 basidiomycetes and two ascomycetes were obtained from JGI Genome Portal (http://genome.jgi-psf.org/). The frequencies of tetranucleotides were calculated by a custom Perl script. CA [33], which is a multivariate analysis method for profile data, was performed against the relative frequencies of tetranucleotides in 23 basidiomycetes including *P* *. porrigens* and two ascomycetes. CA summarizes an originally high-dimensional data matrix [rows (tetranucleotides) and columns (genomes)] into a low-dimensional projection (space) [34–37]. Scores (coordinates) in the low-dimensional space are given to each genome. The distance between plots (genomes) in a low-dimensional space theoretically depends on the degrees of similarity in the relative frequencies of tetranucreotides: a short distance means similar relative frequencies of the tetranucleotides between genomes, whereas a long distance means different relative frequencies. Therefore, distance can be used as an index for similarity among genomes in the relative frequencies of tetranucleotides. Distances between all genome pairs were calculated.

### Functional annotation of unigenes

For functional annotations, sequences of unigenes were searched against the NCBI nr protein database by local BLASTX [38]. The E-value cutoff was set at 1e-5. In the BLASTX searches, the top hit with the highest score at the coverage ≥ 50 was considered as a significant hit for each unigene.

GO [39] analysis was also conducted on unigenes by using InterProScan [40]. The GO terms in biological processes, molecular functions and cellular components were assigned with each unigene. The GO slim terms were also assigned with each unigene according to ontology-related files available from Gene Ontology Consortium (http://www.geneontology.org/GO.downloads.files.shtml).

Gene ortholog assignment and pathway mapping for unigenes were done using KEGG automatic annotation server (KAAS) [41]. By sequence similarity searches against 31 fungi in KEGG database, unigenes were assigned with KO IDs. We used a search method ‘the single-directional best hit information method’ in the KAAS. KO ID represents an ortholog group of genes, which is also directly assigned with information on the KEGG pathways and BRITE functional hierarchy [41,42].

### Read mapping and expression analysis of unigenes

The expression levels of the fruiting bodies and the mycelia for unigene were measured with RPKM values [43]. For RPKM measurement, we mapped the transcriptome reads against unigenes by using BWA (version 0.5.9) [44]. We selected over 50 bp reads which were mapped in a unigene with perfect matches, then counted the number of reads for each unigene. The numbers of reads for each unigene were used to calculate RPKM.

### Semi-quantitative RT-PCR

RT-PCR was performed to validate gene expressions of some unigenes which were detected in this study. For RT-PCR, total RNA from the fruiting bodies and the mycelia were treated with DNase I and purified using RNeasy Mini Kit following the manufacturer’s protocol (Qiagen). About 3.0 µg of purified total RNA from each sample was used for first strand cDNA synthesis using oligo-dT primer and PrimeScript reverse transcriptase (Takara). Equal quantity of first strand cDNA (from 25 ng total RNA) was used for PCR. Actin gene was used as an internal control. Primer sequences and the expected size of the amplified fragments are given in Table 4. Semi-quantitative analysis of the RT-PCR amplified fragments was done by agarose gel electrophoresis.

**Table 4:** Name of proteins, primers used and expected sizes of the RT-PCR products for semi-quantitative RT-PCR.

Protein Name	Forward primer	Reverse primer	Product size (bp)
Actin	GAAAGGATGAAATGAGAAAGC	GTTGACTGGGGATGAAG	204
δ9-fatty acid desaturase protein	TGGCAATCCTACTCCTC	GAGGCCAAGAGAATATGTAAG	1,596
Siderophore biosynthesis regulatory protein	TGGCTCGGCTCGTC	GGCAAGAGAATTGAAGACG	1,194
*Pleurocybella* *porrigens* lectin	ATGTCCATCCCTGCC	AACGGCTTCGAAGAC	411
Xylitol dehydrogenase	CTGCCAGAATCGTAGC	CCAGAAGCGACTAAGG	394
Cyclin	CCTCCATCGTCAAGC	CATCGTCACTCGAGAG	1,539
Glycoside hydrolase family 15 protein	TACACCTGGGTGCGG	GTTCATCGCCACGTATC	1,520
Chitin synthase	GCACTATTGGCGGGAG	GCGAGATACATATTGCGTTC	1,418
Cytochrome P 450	CTCACCAAGACCACTC	CTCTAGGAAATAGCGTCG	1,509
Isocitrate dehydrogenase	AACACTGAAGGAGAGTATTC	GAAGATAGAGGCATCACG	420
Thioredoxin	GCTATCTCATACAATGCCTG	CTTCCTGGGAGATTTGG	221
Eukaryotic initiation factor 4F subunit P130	ATGAGCAAATCTTCGACTGC	CCTTCACTCTCTCAGCC	1,516
Glycosyltransferase family 2 protein	CACCTGTGACCCTGATG	GGTATATTTGCAAACGCTTGG	1,482

## Supporting Information

Figure S1 Comparisons of genome signatures between *P* *. porrigens* and other basidiomycetes and ascomycetes. The distribution of the 23 basidiomycetes and two ascomycetes genomes in the low dimensional space. Although CA provids the scores (coordinates) to the genomes in 24 dimensions, the figure shows in the first two dimensional space. The distances between two genomes were calculated on the basis of scores of all 24 dimensions. For basidiomycetes, the order Agaricals, Tremellales, Russulales, Corticales, Puccinales, Polyporales, Boletales, Gloeophyllales and Auriculariales are shown with red, purple, pink, gray, green, blue, light blue, yellow and black symbol(s), respectively. For ascomycete, Eurotiales and Hypocreales are shown with orange and brown.
(DOC)

Figure S2 The numbers of uniquely mapped read(s) and multi-mapped read(s) to each unigene and the numbers of homolog(s). Reads were classified into uniquely mapped reads and multi-mapped reads according to the mapping results (SAM file). Homologous genes were searched by using BLAST (identity ≥ 90% and coverage ≥ 80%). The asterisks (*) indicate unigenes showing inconsistency in expression levels between RPKM and RT-PCR.
(DOC)

Figure S3 Pathway assignment based on KEGG. The number of unigenes was counted in each BRITE hierarchy. (A) Classification based on primary metabolism categories. (B) Classification based on secondary metabolism categories.
(DOC)

Figure S4 Enzymes involved in the terpenoid backbone based on KEGG. The enzymes that were found in this study were marked by red rectangles. Blue boxes indicate the enzymes that were not found in *P. porrigens*. Mevalonate kinase and farnesyl diphosphate synthase are shown by green circle.
(DOC)

Table S1 **The distances between *Pleurocybella porrigens* and other species (basidiomycetes and ascomycetes)**.
(DOC)

Table S2 **Expression analysis based on reads per kilobase per million (RPKM) values and RT-PCR validation results.**
(DOC)

Table S3 **The numbers of reads mapped to each unigene.**
(DOC)
