# Supplementary material for: A New Omics Data Resource of Pleurocybella porrigens for Gene Discovery
Source: PLoS One. 2013 Jul 23;8(7):e69681. doi: 10.1371/journal.pone.0069681 (PMC3720577; doi:10.1371/journal.pone.0069681)
Supplement: Figure S1 — The distribution of the 23 basidiomycetes and two ascomycetes genomes in the low dimensional space. Although CA provids the scores (coordinates) to the genomes in 24 dimensions, the figure shows in the first two dimensional space. The distances between two genomes were calculated on the basis of scores of all 24 dimensions. For basidiomycetes, the order Agaricals, Tremellales, Russulales, Corticales, Puccinales, Polyporales, Boletales, Gloeophyllales and Auriculariales are shown with red, purple, pink, gray, green, blue, light blue, yellow and black symbol(s), respectively. For ascomycete, Eurotiales and Hypocreales are shown with orange and brown. (DOC) [file pone.0069681.s001.doc]

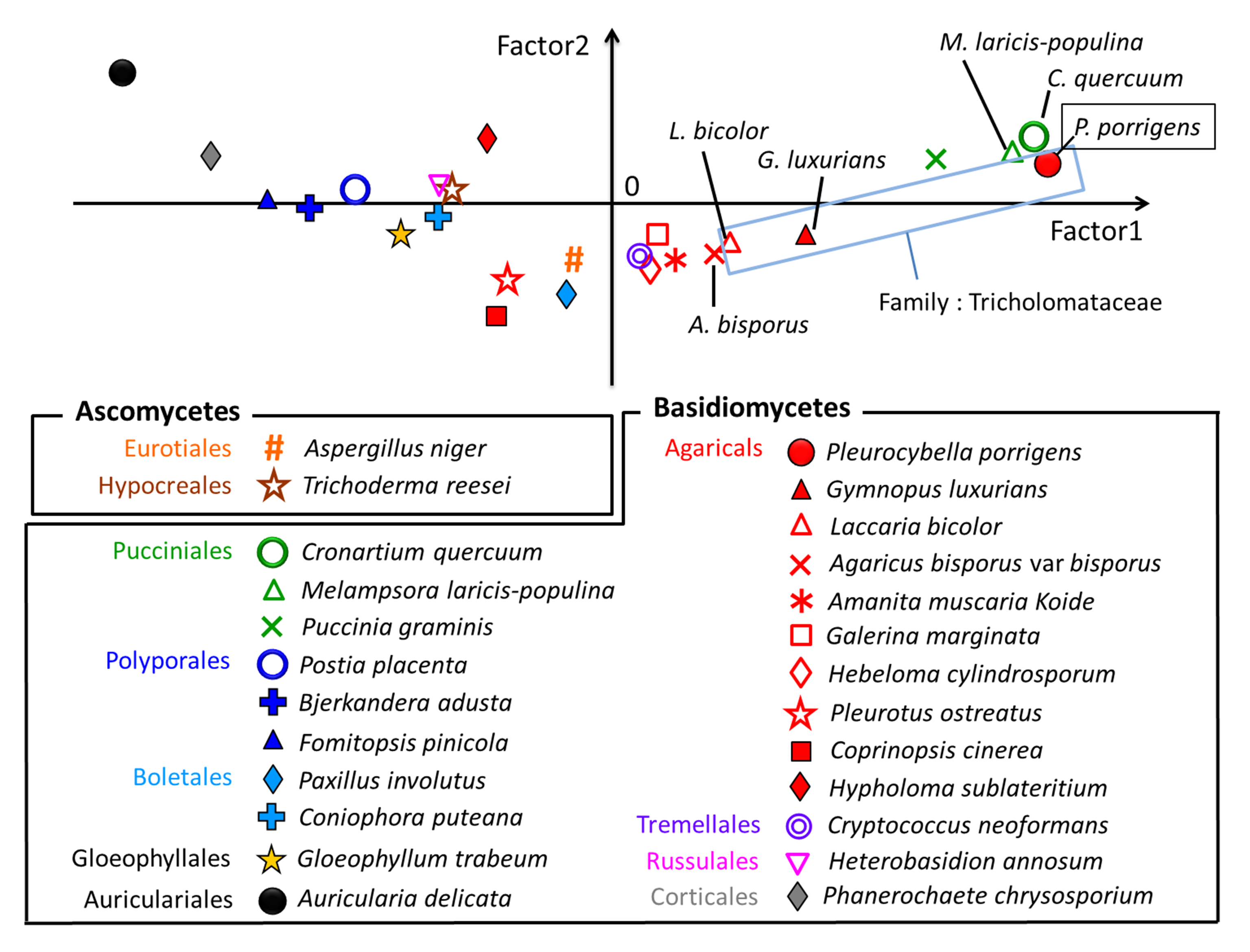


**Figure S1. Comparisons of genome signatures between *P. porrigens* and other basidiomycetes and ascomycetes**. The distribution of the 23 basidiomycetes and two ascomycetes genomes in the low dimensional space. Although CA provids the scores (coordinates) to the genomes in 24 dimensions, the figure shows in the first two dimensional space. The distances between two genomes were calculated on the basis of scores of all 24 dimensions. For basidiomycetes, the order Agaricals, Tremellales, Russulales, Corticales, Puccinales, Polyporales, Boletales, Gloeophyllales and Auriculariales are shown with red, purple, pink, gray, green, blue, light blue, yellow and black symbol(s), respectively. For ascomycete, Eurotiales and Hypocreales are shown with orange and brown.
