# Supplementary material for: A New Omics Data Resource of Pleurocybella porrigens for Gene Discovery
Source: PLoS One. 2013 Jul 23;8(7):e69681. doi: 10.1371/journal.pone.0069681 (PMC3720577; doi:10.1371/journal.pone.0069681)
Supplement: Figure S2 — Reads were classified into uniquely mapped reads and multi-mapped reads according to the mapping results (SAM file). Homologous genes were searched by using BLAST (identity ≥ 90% and coverage ≥ 80%). The asterisks (*) indicate unigenes showing inconsistency in expression levels between RPKM and RT-PCR. (DOC) [file pone.0069681.s002.doc]

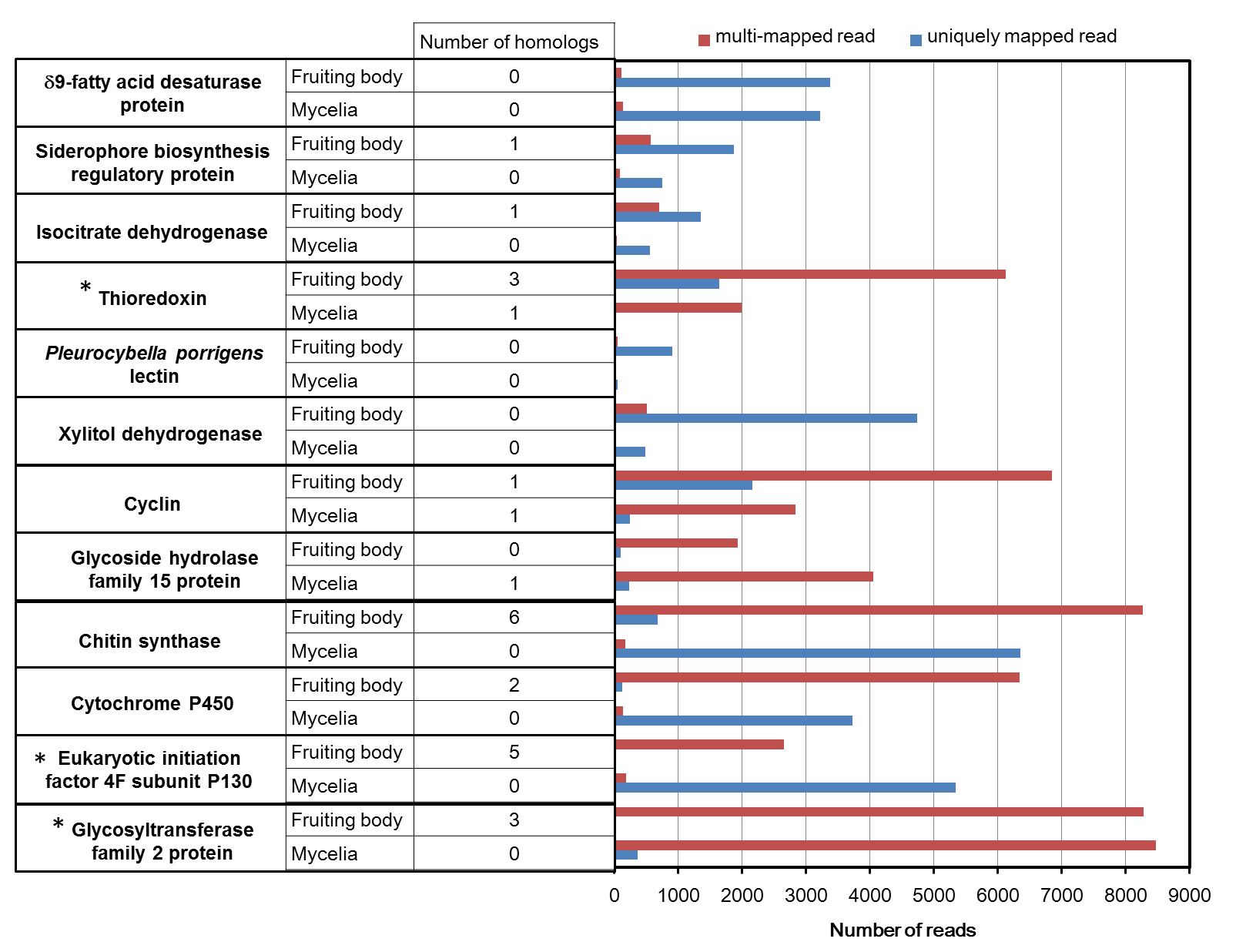


**Figure S2. The numbers of uniquely mapped read(s) and multi-mapped read(s) to each unigene and the numbers of homolog(s).** Reads were classified into uniquely mapped reads and multi-mapped reads according to the mapping results (SAM file). Homologous genes were searched by using BLAST (identity ≥ 90% and coverage ≥ 80%). The asterisks (*) indicate unigenes showing inconsistency in expression levels between RPKM and RT-PCR.
