# Supplementary material for: A New Omics Data Resource of Pleurocybella porrigens for Gene Discovery
Source: PLoS One. 2013 Jul 23;8(7):e69681. doi: 10.1371/journal.pone.0069681 (PMC3720577; doi:10.1371/journal.pone.0069681)
Supplement: Figure S3 — The number of unigenes was counted in each BRITE hierarchy. (A) Classification based on primary metabolism categories. (B) Classification based on secondary metabolism categories. (DOC) [file pone.0069681.s003.doc]

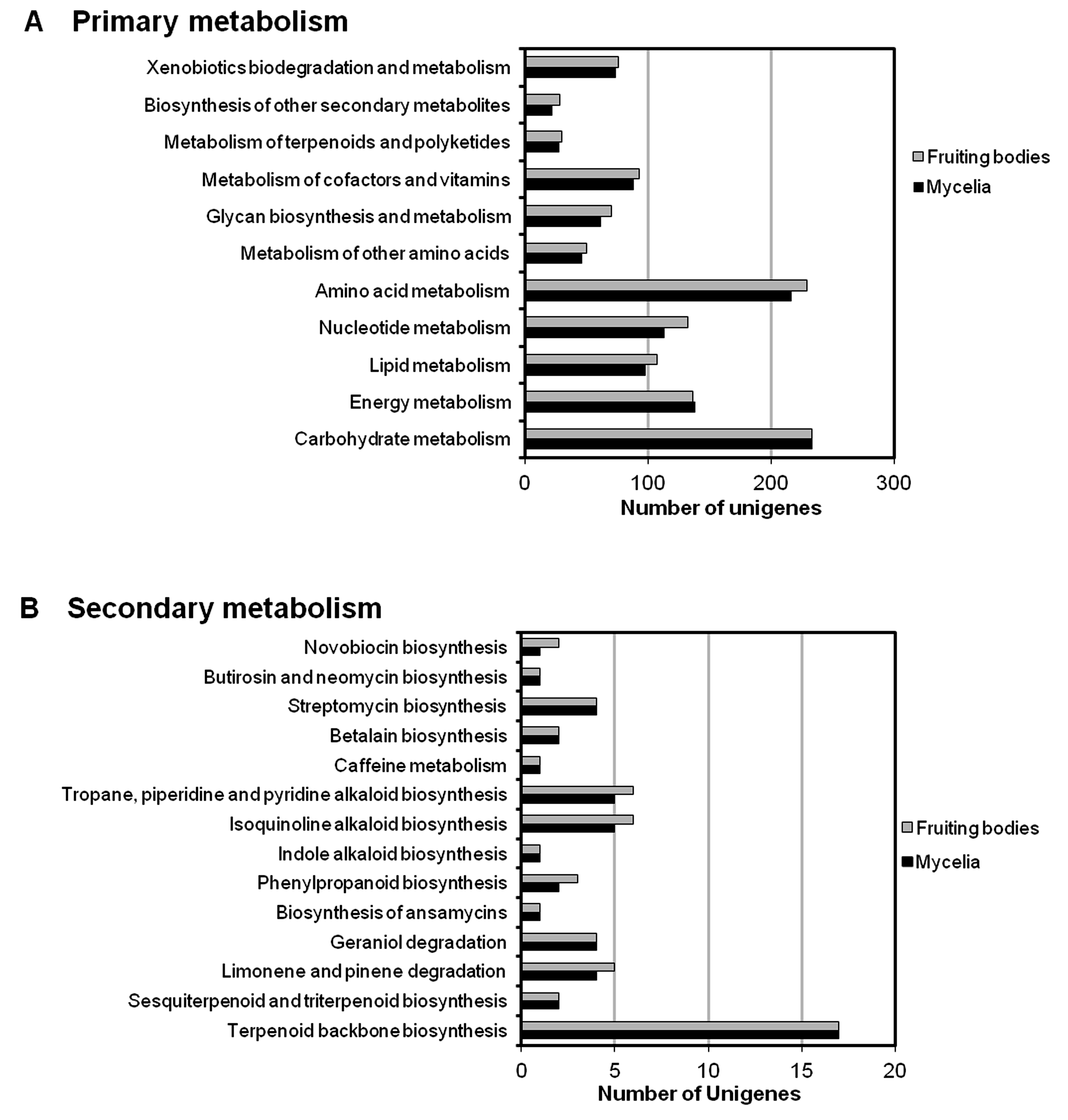


**Figure S3. Pathway assignment based on KEGG.** The number of unigenes was counted in each BRITE hierarchy. (A) Classification based on primary metabolism categories. (B) Classification based on secondary metabolism categories.
