# Supplementary material for: A New Omics Data Resource of Pleurocybella porrigens for Gene Discovery
Source: PLoS One. 2013 Jul 23;8(7):e69681. doi: 10.1371/journal.pone.0069681 (PMC3720577; doi:10.1371/journal.pone.0069681)
Supplement: Figure S4 — The enzymes that were found in this study were marked by red rectangles. Blue boxes indicate the enzymes that were not found in P. porrigens. Mevalonate kinase and farnesyl diphosphate synthase are shown by green circle. (DOC) [file pone.0069681.s004.doc]

**
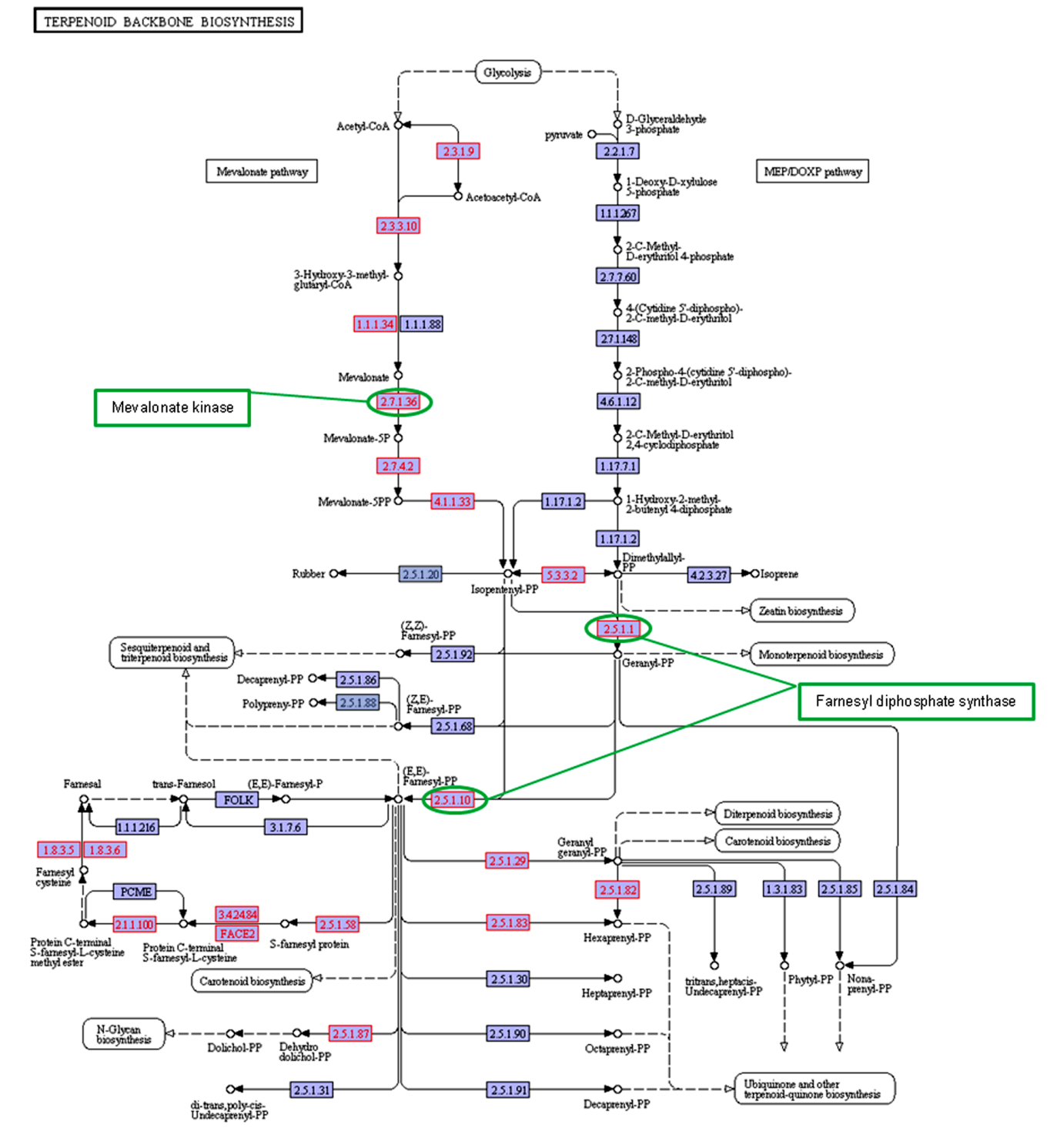
**

**Figure S4.** **Enzymes involved in the terpenoid backbone based on KEGG.** The enzymes that were found in this study were marked by red rectangles. Blue boxes indicate the enzymes that were not found in *P. porrigens*. Mevalonate kinase and farnesyl diphosphate synthase are shown by green circle.
