# Supplementary material for: A New Omics Data Resource of Pleurocybella porrigens for Gene Discovery
Source: PLoS One. 2013 Jul 23;8(7):e69681. doi: 10.1371/journal.pone.0069681 (PMC3720577; doi:10.1371/journal.pone.0069681)
Supplement: Table S1 — The distances between Pleurocybella porrigens and other species (basidiomycetes and ascomycetes). (DOC) [file pone.0069681.s005.doc]

**Table S1.**

The distances between *Pleurocybella porrigens*

and other species (basidiomycetes and ascomycetes).

| Fungal species | Distances |
| --- | --- |
| *Cronartium quercuum* | 0.235 |
| *Melampsora laricis-populina* | 0.247 |
| *Gymnopus luxurians* | 0.282 |
| *Puccinia graminis* | 0.291 |
| *Agaricus bisporus* var *bisporus* | 0.358 |
| *Laccaria bicolor* | 0.364 |
| *Amanita muscaria Koide* | 0.388 |
| *Galerina marginata* | 0.403 |
| *Cryptococcus neoformans* | 0.422 |
| *Hebeloma cylindrosporum* | 0.438 |
| *Aspergillus niger* | 0.471 |
| *Paxillus involutus* | 0.486 |
| *Pleurotus ostreatus* | 0.516 |
| *Hypholoma sublateritium* | 0.517 |
| *Coprinopsis cinerea* | 0.559 |
| *Heterobasidion annosum* | 0.562 |
| *Coniophora puteana* | 0.568 |
| *Trichoderma reesei* | 0.573 |
| *Gloeophyllum trabeum* | 0.593 |
| *Postia placenta* | 0.629 |
| *Bjerkandera adusta* | 0.679 |
| *Fomitopsis pinicola* | 0.706 |
| *Phanerochaete chrysosporium* | 0.752 |
| *Auricularia delicata* | 0.834 |
