# Supplementary material for: A New Omics Data Resource of Pleurocybella porrigens for Gene Discovery
Source: PLoS One. 2013 Jul 23;8(7):e69681. doi: 10.1371/journal.pone.0069681 (PMC3720577; doi:10.1371/journal.pone.0069681)
Supplement: Table S2 — Expression analysis based on reads per kilobase per million (RPKM) values and RT-PCR validation results. (DOC) [file pone.0069681.s006.doc]

**Table S2.** Expression analysis based on reads per kilobase per million (RPKM) values

and RT-PCR validation results.

| 1. **The unigenes with similar expression levels between fruiting bodies and mycelia** | | | |
| --- | --- | --- | --- |
| Unigene | Fruiting bodies | Mycelia | Ratio (F/M)* |
| δ9-fatty acid desaturase protein | 121.31 | 184.18 | 0.7 |
| Siderophore biosynthesis regulatory protein | 82.11 | 46.75 | 1.8 |
| Isocitrate dehydrogenase | 78.19 | 61.94 | 1.3 |
| Thioredoxin ** | 284.14 | 164.34 | 1.7 |
|  | | | |
| 1. **The unigenes with high expression levels in fruiting bodies** | | | |
| Unigene | Fruiting bodies | Mycelia | Ratio (F/M)* |
| *Pleurocybella porrigens* lectin | 62.3 | 7.32 | 8.5 |
| Xylitol dehydrogenase | 293.89 | 65.06 | 4.5 |
| Cyclin | 107.08 | 54.06 | 2.0 |
|  | | | |
| 1. **The unigenes with low expression levels in fruiting bodies** | | | |
| Unigene | Fruiting bodies | Mycelia | Ratio (F/M)* |
| Glycosidase family 15 protein | 29.07 | 91.31 | 0.3 |
| Chitin synthase | 78.01 | 318.19 | 0.2 |
| Cytochrome P 450 | 55.6 | 196.01 | 0.3 |
| Eukaryotic initiation factor 4F subunit P130 ** | 27.01 | 241.64 | 0.1 |
| Glycosyltransferase family 2 protein ** | 47.57 | 134.36 | 0.4 |
| The ratio between 0.5 – 2.0 is considered as the similar expression level.  * means RPKM ratios between the fruiting bodies and the mycelia.  ** signifies the inconsistency in expression levels between RPKM and RT-PCR. | | | |
