# Supplementary material for: A New Omics Data Resource of Pleurocybella porrigens for Gene Discovery
Source: PLoS One. 2013 Jul 23;8(7):e69681. doi: 10.1371/journal.pone.0069681 (PMC3720577; doi:10.1371/journal.pone.0069681)
Supplement: Table S3 — The numbers of reads mapped to each unigene. (DOC) [file pone.0069681.s007.doc]

**Table S3 .** The numbers of reads mapped to each unigene.

| 1. **The numbers of transcriptome reads mapped into a unigene.*** | | |
| --- | --- | --- |
| Summary of the numbers of reads | Fruiting bodies | Mycelia |
| Range | 1 - 17,610 | 1 - 10,253 |
| Median | 58 | 31 |
| Average | 227 | 214 |
|  |  |  |
| 1. **The numbers of transcriptome reads mapped into a unigene showing the same expression levels in fruiting bodies and mycelia.*** | | |
| Unigene | Fruiting bodies | Mycelia |
| δ9-fatty acid desaturase protein | 1,940 | 1,626 |
| Siderophore biosynthesis regulatory protein | 1,055 | 367 |
| Isocitrate dehydrogenase | 885 | 245 |
| Thioredoxin ** | 664 | 330 |
|  |  |  |
| 1. **The numbers of transcriptome reads mapped into a unigene showing the high expression levels in fruiting bodies.*** | | |
| Unigene | Fruiting bodies | Mycelia |
| *Pleurocybella porrigens* lectin | 429 | 17 |
| Xylitol dehydrogenase | 2,343 | 235 |
| Cyclin | 2,423 | 672 |
|  |  |  |
| 1. **The numbers of transcriptome reads mapped into a unigene showing the low expression levels in fruiting bodies.*** | | |
| Unigene | Fruiting bodies | Mycelia |
| Glycosidase family 15 protein | 549 | 995 |
| Chitin synthase | 1,787 | 2,700 |
| Cytochrome P 450 | 925 | 1,737 |
| Eukaryotic initiation factor 4F subunit P130 ** | 438 | 2,386 |
| Glycosyltransferase family 2 protein ** | 1,242 | 2,012 |
| * Transcriptome reads from fruiting bodies and mycelia were mapped into unigenes from fruiting bodies and mycelia, respectively. Then, the numbers of transcriptome reads mapped into unigenes were counted.  ** signifies the inconsistency in expression levels between RPKM and RT-PCR. | | |
